# A narrative analysis of learning to teamwork in youth sport: a sociocultural perspective on communication and cooperative learning

**DOI:** 10.3389/fspor.2025.1667534

**Published:** 2025-11-11

**Authors:** Joan Arumí-Prat, Gil Pla-Campas

**Affiliations:** Sport, Exercise, and Human Movement Research Group, University of Vic – Central University of Catalonia, Vic, Spain

**Keywords:** narrative research, communication, team sport, basketball, coaching approach, sociocultural perspective, autoethnography, teamwork

## Abstract

This study investigates from a sociocultural perspective how team members' communication within a training approach based on cooperative learning (CL) fosters the development of teamwork. Employing a narrative analytic approach, the research examines into the experiences of a coach and his players in the locker room conversations before, during and after the game, throughout the season. A total of 69 5- to 17-minute conversations were recorded and transcribed over 23 matches that took place during an entire season. Three different steps were implemented for the narrative analysis: codification of the dialogues under the CL approach, identification of stages of learning in players and creation of the autoethnographic storytelling. Findings are shown through a narrative based on five significant stories that illustrate the context, and the complex dynamics of social interaction that emerge to learn teamwork in youth sport are discussed. By examining the coach's adoption of CL principles as a coaching and communication approach between team members, this research shows how these principles frame the learning context and contribute to teamwork. This research reveals the complexities of player-coach interactions and provides valuable insights for youth team sport coaches committed to fostering teamwork.

## Introduction

As Lyle (1) suggests, coaching is an uncertain, complex and singular process that defies simple analysis. McEwan and Beauchamp (2) consider that coaching should be tailored to each team rather than a “one-size-fits-all” approach. Furthermore, the role of a youth sport coach is complex and multidimensional that can include the roles of teacher, trainer, motivator, disciplinarian or a friend, among others (3). These features extend to the intricate dynamics of educational interaction within coaching settings and underscore the need for effective communication, a sentiment echoed by Martens (4), who suggests that “coaching is all about communication” (p. 96).

Building upon Lyle's insights into the singular nature of coaching and Martens' assertion about the pivotal role of communication in navigating the complex nature of coaching, this research aims to explore, from an autoethnographic point of view and with a sociocultural perspective (5), the communication aids a coach uses to foster teamwork in a team of 13-year-old, male basketball players and within a cooperative learning coaching approach. To do this, the research uses a narrative analytic method (6) that, unlike other scientific approaches, allows us to capture the singular circumstances in which learning teamwork takes place through the communication between coach and athletes in the hidden and protected coaching context of locker room conversations before, during and after the game.

### The didactic strategy of cooperative learning to build teamwork

Cooperative Learning, as both a pedagogical and sociocultural approach rooted in the understanding of education as a social process (7), is widely used in schools. It consists of the didactic use of small teams of students in which the structure of the activity (8) is used to maximise equitable participation and simultaneous interaction between them. The purpose of this educational intervention approach is for all members of a team to learn the content that the context demands of them, do their best and learn to work as a team (8, 9). This research acknowledges that teamwork and CL are not the same, but it recognises that CL is a specific didactic structure to develop teamwork.

According to Johnson et al. (8), CL requires the presence of five basic conditions that must be present in the learning activities: a) *Positive interdependence*. Each group member learns to depend on the rest of the group as they work together to complete a task. b) *Individual accountability*. Establishing each student's responsibility for appropriate behaviour, involvement in the task and goal achievement. c) *Face-to-face promotive interaction*. Encouraging and facilitating mutual effort to produce, complete and accomplish tasks in order to achieve the objectives of the group. d) *Social skills*. Using and learning interpersonal and small-group skills for high-quality collaboration between students. e) *Group processing*. The effectiveness of group work is influenced by whether or not the groups reflect on how they work. These five basic conditions frame the foundation upon which the communication strategies and design of tasks of the cooperative approach are based.

The same five basic conditions that Johnson et al. (8) proposed have been used in the field of physical education and physical activity (10, 11), and CL is considered one of the pedagogical models of the future in physical education (12). There is empirical evidence to show that CL has advantages over traditional methodologies based on individual and competitive pedagogical approaches. Slavin (13) shows the positive effects of CL on motivation, social cohesion, development and cognitive elaboration. In the application of physical education programmes using CL, Casey and Goodyear (14) conclude that students improve physical, cognitive and social domains, as well as increasing academic performance by understanding and applying the educational content. Other authors coincide with this, suggest activities and highlight the improvement in motor, tactical or strategic skills that learners can achieve through CL (10, 14). Therefore, a CL coaching approach could promote not only the development of the skills required for teamwork but also the specific playing motor skills.

Teachers and coaches often assume that if they teach a cooperative activity, the students or athletes will automatically learn interpersonal skills in small groups, but this is not the case (10). Learning to work in a team requires time and strategies that are well-defined and powerful, such as those proposed by Johnson et al. (8), Kagan (9) and Slavin (13), but it goes beyond cooperative techniques as it is a communicative process built through the interaction of all team members. Teamwork is a collective process that involves variables of collective dynamics such as cohesion, team conflict, collective efficacy and transactive memory (15).

### Communication as the mediator of teamwork learning

The social, cultural, linguistic and longitudinal nature of this research leads us to observe the phenomenon under study from a sociocultural perspective that places the interaction between people, in both its individual and collective dimension, in the context that drives learning and development (5). This approach will allow us, as Moen (16) points out, to make explicit the epistemological connection inherent in human phenomena in which individual and social, and therefore also historical and cultural, processes are mixed.

From a sociocultural perspective, learning, which in this research is the meaning of teamwork, is due to the help that an expert (the coach) provides to a learner (the athletes of the team). The athletes access this learning through pedagogical aids, organised by the coach with more or less conscious decisions. This organisation entails the generation of an educational intervention, which takes place within the well-known Zone of Proximal Development (ZPD) (17). According to Vygotsky (17), two types of aids mediate between the expert and the learner: tools and signs. While tools represent material artefacts, signs are the psychological aids that culture provides and, according to Cole (18), language is the most powerful cultural aid available to promote learning and, therefore, to transform human behaviour. Thanks to these aids, which mediate between the subjects themselves and the world, the expert provides means to promote their learning. In other words, the coach's notion of teamwork precedes that of the athletes, and through language, which is one of the mediators of the culture, the coach can promote the athlete's learning process. The research, therefore, places language, in the form of conversations, dialogues, discussion and comments, as the specifically human pedagogical support (17) that mediates in the athletes learning.

The sociocultural approach shows us that access to new behaviours is not an individual process but a social and interactional one. This is called the process of internalisation and is defined as the internal reconstruction of an external psychological operation (17). In the same vein, Edwards and Mercer (19) view education as a process of communication and emphasise the content, meaning and context of discourse to examine how shared knowledge is established. However, the interplay between the coach and athletes is epistemologically a relationship that involves the personal transformation of both sides of the dialogue. This is why Cole (18) states that the cultural transmission of learning processes takes place intersubjectively. That is, the inherent nature of dialectical materialism leads to cultural change that affects not only the learner but also the teacher. In this research, the internal operation which is under study is the meaning of teamwork, which is based on individual players who understand the “objectives” and “functions” of themselves that might positively impact to the team performance (20).

Therefore, considering a team as a cultural context itself, encompassing the interactions of everyday training and matches and the temporal evolution of both the team and its members, echoes Cole's concept of cultural scripts (18). Building upon this notion, Bruner (21) suggests that we can understand these dynamics through narrative by examining events, their sequence and the connections between them. This highlights the role of narratives as a means to make sense of the world, something that the stories of this investigation will allow us to do. Thus, narrative serves as an ideal sociocultural lens for elucidating the process of learning to build teamwork through conversational exchanges during matches.

## Materials and methods

This research is situated in the constructivist paradigm, which presupposes a relativistic ontology and subjectivist epistemology in which the researcher and the researched jointly create the meaning of “teamwork” through communication that is the product of the communicative interaction between the expert and the learners (17). Along with the theoretical background, this narrative is underpinned by a sociocultural approach capable of showing how communication emerges and evolves from social relationships, not from individual minds, and emphasizes the changing reality of human beings as meaning-makers (6). The dialogues between the coach James (expert) and the players (learners) are placed at the centre of the research since they are the mediators of interaction that will help explain the development of teamwork skills in the young players.

### Sample and context

The study was carried out on a basketball team of eleven 13-year-old boys at the beginning of the season, with at least five years of basketball training experience. This team competed in a medium to low-level regional category organised by a regional Federation in a country where basketball is the sport with the second largest number of licences and has achieved very significant international success. The team trained three times per week and played one game per week. Technical, tactical and strategic motor skills training were scheduled, sequenced and cognitively mobilised in the team through CL techniques tailored to team sport coaching. The players were immersed in these cooperative techniques for the first time in basketball training. Multiple and varied CL techniques were used in training and on match days, for example: Elliot Aronson's “Jigsaw Puzzle”, Spencer Kagan's “Time Pair Share” and Ben Dyson's “Learning Team”. But at the same time, the discussion among team members that coach James fostered followed a CL approach to communication to seek a positive emergence of the CL categories in the language of the players.

### Research design

With the aim of revealing the communication aids a coach uses to build teamwork through CL approach in a youth basketball team, both a story analysis and storytelling are used. As Smith (6) states, the family of narrative analytic methods can be sorted into two different standpoints towards stories: the story analyst and storyteller. Neither one is better than the other, and researchers may choose to operate as one or the other, moving back and forth between standpoints. Likewise, there are different types of narrative analysis, and the one this research is based on is thematic narrative analysis, which, in the words of Braun and Clarke (22), is a “*fully* qualitative” (p. 2) flexible method. Braun and Clarke consider that thematic analysis requires continually bending back on oneself, questioning and querying the assumptions made in the interpretation process.

In parallel with the thematic analysis, a story is written by means of an autoethnography, which is a type of *Creative Analytical Practice* (CAP) (6). Some have considered the tensions that this type of research brings to light (23), but we believe that these research approaches are equally necessary to understand the complexity involved in coaching. CAPs enable researchers to show a reflexive, partial, contextual and constantly changing reality (24) and, accordingly, this research attempts to show the unforeseen and intricate dialogue complexities involved in building teamwork, which are no less real or worthy. In words of Richardson and Pierre (25), the CAPs are also considered to be “*both* creative *and* analytic” (p. 930) and, aligned with Sparkes (26) proposal for autoethnographies, the storyline written as a result of our research process is an analytic autoethnography with landscapes of *evocative autoethnography*. In this regard, the autoethnographic storyline aims, as some authors argue, to create a story that supports a theory (27). Such a narrative with an autoethnographic representation has already been used by other authors in the field of sport coaching (28–30) and CL in physical education (31).

James, as the principal investigator of the research, presented the aims of the research in the first meeting of the season with the athletes' parents. After this, parents provided informed consent prior to participation, and confidentiality and data protection regulations were strictly observed. Likewise, the athletes were informed about the intentions and methods of the research at the beginning of the season, and they all agreed to take part in this research. In accordance with the purpose of the research, all the conversations between the coach and team before the match, during half-time and after the match were recorded. A total of 69 5- to 17-minute conversations were recorded over 23 matches that took place between September and May. Verbatim transcripts of the conversation were made directly from the recording and by a single transcriber.

Three sequential steps were implemented in this narrative analysis:

Step 1. Coding the dialogues. The codification followed a deductive process of defining the unit of analysis and subsequent categorisation. Eight hundred and ninety-eight sentences were identified for the narrative analysis. The units of analysis were defined as the phrases or statements that the athletes made during the talks before, during and after the match, and these units were codified in relation to the five basic conditions of CL outlined by Johnson et al. (8). The coding process aimed to find units of analysis that described the expression of each CL category in its positive form and whether the units appeared showing the negative or opposite expression or simply did not appear. In the Table 1, we show examples of what we defined as a sentence and examples of its positive or negative codification in relation to the CL categories:
Step 2. Analysis of the evolution of athlete's sentences throughout the season. With the lens of the CL categories, an inductive process was followed to identify transition stages in the players' understanding of teamwork in relation to the communication strategies taken by the coach. According to this, three main periods were identified:
  - Period 1. Identification of the athlete's meaning of teamwork. The coach aims to foster an open and reflective environment that showcases the challenges faced in promoting teamwork.
  - Period 2. Internalisation aids of teamwork within the ZPD. The coach opens conversation topics of interest, sets boundaries and narrows conversations to enhance a positive conversational setting designed to shape a better meaning of teamwork.
  - Period 3. Improvements in the meaning of teamwork. The coach again promotes open dialogues to reflect upon the team's performance. The improvements in the player's understanding of teamwork in conversations showcase the internalization process they went through.
Step 3. Autoethnographic storytelling creation. By using this storytelling approach, the study follows (32) call for sport psychology research to embrace CAPs that highlight lived experiences and real-world complexities in a way that traditional research formats often fail to capture. Therefore, of the set of 69 recorded conversations, James wrote the autoethnographic narrative of the learning to teamwork in his U14 youth team through different stories. They express procedural temporality, social interaction and context of the events, elements that Clandinin et al. (33) consider a narrative must have.

**Table 1:** Codification.

Categories	Examples of athlete's sentences (as units of analysis)
Positive interdependence	“If I catch the rebound, we can play the counterattack.”
Individual accountability	Positive: “I defended the number 5.” Negative: “I don't want to defend”
Face-to-face promotive interaction	Positive: “I will support Rob if he misses passes.” Negative: “he can't stop the player”
Social skills	Negative: “Kill the referee.”
Group processing	“I'm sorry, I failed in the defensive balance.”

*Source:* Authors.

## Results

The results of this research are presented in five stories that show the conversational interactions between the players and coach during throughout the season and changes in communication to show the periods of the conversations. In addition to this, the stories were selected based on the appearance of the five basic conditions of CL, as others have done (31), to highlight how teamwork in sport and a cooperative coaching approach are linked. In the following sections, the coach's thoughts and the team's conversations are presented in italics, while the analytical discussion of their content is provided in regular font.

The first story, entitled “How did the game go?”, depicts James, the coach in charge of the group, entering the locker room after the first basketball game of the competition in mid-September, seeking positive reply from the players about the game. The coach aims to foster an open and reflective environment, but the players' responses take an unexpected turn for him. The conversation revolves around one of the conditions of CL: group processing (8).

### Story 1. How did the game go?

*As soon as I entered the locker room, I let out a “cool” question. I thought it was a good way to introduce my new coaching style. I wanted us all to reflect on what had happened in an uninhibited, carefree way and to talk about the improvements we could make for the next game. I was calm because we had won, but I immediately noticed that my body was tense:*

*James: Eh, how did the game go?*

*Brian: Bad.*

*James: Why bad?*

*Brian: Bad mate, we should have won by more than 50 points.*

*James: Don't you think that's a little arrogant?*

*Brian: Can we have a go at the referee?*

*James: The referee has no excuse, and I don't plan to get involved.*

*Brian: Goddamn it.*

*Oscar: We'll have a go at him.*

*I couldn't believe what I was feeling. Brian had immediately set an aggressive tone to the conversation and I, disoriented, had never imagined that this could happen to me. So many moments to think about my coaching style to let out a trusting: how did the game go? And without anticipating it, unleash all that reaction. It had got all out of hand and I didn't know how to stop it. It was a mess, and the snowball was picking up speed.*

*James: Wait a moment …* *If you raise your hand, I'll give you the floor.*

*Cooper: Yes, but if they do a foul and the referee goes against us.*

*James: Cooper no, no…*

*Hayden: If they commit a foul, I'll react.*

*James: Hayden reacts, great. You should know that we have to change, that we can't go along this path, ok?*

*Cooper: The referee let them get away with loads of fouls, loads of them.*

*Kurt: The referee was bribed.*

*James: But …* *we also make mistakes, right? Well, referees too.*

*Oscar: There was one in the other team that…*

*Brian: That was a total fagot.*

*James: Come on!*

*Rob: 13 was a bastard.*

*James: Don't insult. Enough, please.*

*I kept my cool and didn't raise my voice. I gathered them in the middle of the locker room, and we shouted the battle cry: 1, 2, 3 Team! I picked up my things from the locker room to leave while feeling completely disoriented. I didn't know what had happened, but now I had the answer to why my former coaches didn't let us open our mouths. I left the locker room and closed the door behind me.*

The coach engages players in discussions about the game, creating a social context to uncover the meaning of teamwork that players have. Teachers and coaches often assume that a cooperative learning setting implies automatically the learning of interpersonal skills, but this is not the case (10). We have seen that the dialogues illustrate a dynamic and inappropriate interaction between the coach and players, with a negative team reaction to his initial question: How did *the game go?* As a first analysis, the conversation shows that the players failed to identify their own behaviour on the court or acknowledge their own mistakes. They did not recognise the functions and objectives (20) of an individual with respect to teamwork. This situation reveals what Leo et al. (15) have pointed out, which is that team conflicts arise when coaches foster teamwork. The non-identification of one's own mistakes was expressed by the lack of face-to-face promotive interaction (they do not praise but rather disdain the referee and rivals) and the lack of positive social skills (they do not listen or wait for their turn to speak) which correspond to two of the five cooperative learning conditions described by Johnson et al. (8)

In this first story, we find a coach that as a young player had never been asked about the game because his coaches were all authoritarian, but he nevertheless takes the CL approach in coaching believing that players know what teamwork means and expecting they will behave accordingly. The players' answers to the questions catch the coach off guard because these answers are completely unexpected, therefore uncertain and singular (1) and are not aligned with the meaning of teamwork that he is seeking to promote. This is a signal that an internalisation process (17) to improve the players' meaning of teamwork is required. He could have acted in an authoritarian manner, just as his former coaches did in his youth, but he took the deliberate decision to maintain a CL approach and act in the role of educator (3), which demands temperance to accept and hear diverse voices in a locker room.

The communication of the players at the beginning of the season unsettled the coach because the basic conditions of CL which indicate good teamwork did not emerge in a positive form. The following story took place on January 14 when the team was four months into the season, had played eight competition matches and the dynamics of the locker room conversations were still similar. We have entitled the story: *Where is the cooperation? I look for it, but I can't find it*. It shows the coach's surprise when he realises that the meaning of teamwork does not appear just by using the CL categories as a mean to converse with players.

### Story 2. Where is the cooperation? I look for it, but I can't find it

*My head keeps thinking about activities and making decisions to encourage cooperation: what if I make small groups, what about heterogenous groups, what about discussing the team rules, what about interviewing a colleague, what about this, what about that. I get everything scheduled, everything ready, everything in detail, and then I come across things I didn't expect. But what unsettles me most is when everything I seek turns upside down. Like that day when we lost, and they got into a fight with the referee while I tried to redress the situation. We had lost and no sooner had the talk begun than the boys blamed the referee for the defeat. Luckily Rob, the most mature, helped me make a point:*

*Rob: But it wasn't the referee's fault. We got lost.*

*James: I support you somewhat regarding the referee as he was occasionally wrong.*

*Brian: I mean, you can't play like this.*

*Larry: Let him blow the whistle at the U10.*

*James: Do you know what self-criticism is?*

*Brian: Kill the referee.*

*James: Killing the referee is not an option. What could we have done better?*

*Larry: The referee went too far.*

*Cooper: We did everything right.*

*James: We did everything right? So why did we lose the game?*

*Rob: We missed many passes that were easy.*

*Brian: In the end, after so many hits you get fed up.*

*Where is the cooperation in all this? Arrogance, revenge, disdain. All my careful training schedule, all my cooperative activities designed from good pedagogical books are dismantled through insults and threats. I am so disoriented that I don't know who I am or where I am going. Perhaps I need to open my ears properly, listen to them. Cooperation may not be within me, in my activities, in my exercises. Perhaps cooperation lies outside, with them, in their chatter, in their desire to say what they think, in their spontaneity.*

*Rob's words resonate with me. He, in the midst of a sea of reproaches, says: “But it wasn't the referee's fault. We got lost”. And later he says: “We missed many passes that were…”. His teammates don't let him finish, but his discourse drifts to where I want him to go. I hope that these words land, settle, that the boys start reflecting, start becoming aware of working in a team.*

The coach expresses the internal struggle of trying to encourage cooperation. There is a sense of frustration and confusion because his coaching strategies do not seem to lead to the desired outcomes on the court. Therefore, the training results emerge as uncertain and complex (1). Despite careful planning, the coach encounters unexpected challenges, such as the players blaming the referee after a loss or their lack of self-criticism, which show a lack of individual accountability (8), a lack of individual understanding of the individual “functions” when playing as a team (20). This situation disrupts the coach's efforts to address the team's conversation constructively and his role as coach. James's role as coach is therefore complex and multidimensional (3). That is why we place this conversation in period 1 (Identification of the meaning of teamwork) because the unforeseen dialogue catches the coach off guard, who is therefore still uncovering the players' understanding of teamwork. In summary, the two stories of period 1 examine a common problem faced by coaches in team sport: the different meaning of teamwork that players have in relation to a coach and how this disrupts the learning scenario.

In the following stories, we observe the reaction of the coach to this learning scenario. The coach becomes aware of the need to change the discourse of his players and focus on communication strategies that promote the appearance of the basic conditions of CL. To do so, he establishes clearer guidelines for maintaining a positive communication atmosphere in the discussions before, during and after the game. On the one hand, the discussions involve all individuals going through the process of internalising teamwork as they embody the social nature of human beings as meaning-makers (6). On the other hand, the guidelines are crucial for defining the learning zone in which players can develop and can be set through the communication. In the third story, situated in period 2 (internalisation aids within ZPD), we can see two examples of guiding a dialogue to encourage positive and respectful communication between players. In this case, we will see two conversations held before two games, which denote the presence of the categories of positive interdependence and face-to-face promotive interaction.

### Story 3. Teamwork!

*James: Let's do an activity that I've called “I depend on you”. You have to say to a partner that to do an action you depend on him or vice versa. Who starts?*

*Rob: If I catch the rebound, we can play the counterattack.*

*Brian: If I do a bad pass, Steve won't be able to score points.*

*Steve: If I catch rebounds, Brian will be able to score points.*

*Kurt: If Hayden makes a good cut, I will be able to pass him the ball to score a point.*

*Hayden: If I go to help, perhaps another will come to the defence.*

*Xavier: If I throw myself on the ground to get the ball back, I could pass it to Larry or Oscar.*

*Larry: If I open on the wing, I can pass it to Xavier so that he can go to the basket.*

*I'm building a team by connecting the players. The technique and tactics of basketball help me, as if they were the threads to weave a piece of clothing. We continue playing matches and today I propose another activity to bolster their spirits.*

*James: In today's game you have to encourage each other, and so you'll tell a teammate when you will support him. Who starts?*

*David: I will support Larry if he loses the ball when they defend very strongly.*

*Larry: I will support Rob if he misses passes.*

*Rob: I will support Kurt if they block him.*

*Kurt: I will support Oscar if he makes a bad shot.*

In this story, the coach introduces the “I depend on you” cooperative learning technique that works as an aid (17) to encourage players to recognise their individual function (20) within teamwork during the game. We observe the coach letting the conversation evolve in a positive atmosphere, while players challenge their own old meaning of teamwork. In this case, James points to the positive interdependence (8) category of the CL strategy, and we can observe three different coaching actions: 1) focusing on the topic of interdependence; 2) narrowing the sort of statements the conversation can have; and 3) giving precise examples of sentences to show a positive teamwork meaning. This set of strategies fosters the internalisation of the coach's meaning of teamwork and helps to define a better learning zone (17). Other learning techniques like this one can describe period 2 and were designed not only to foster positive team communication but also to improve specific technical and tactical playing skills.

The next story shows some improvements in the team's meaning of teamwork, but we still consider this story part of period 2 because it focuses on the communication strategies set by the coach. This story, which takes place at the end of March, reveals a change in the behaviour of one of the players looking James in the eyes.

### Story 4. The spark in the eyes

*The five players that were on the court are sitting on the bench for half-time. They are talking to each other but I'm not listening. All five are panting and recovering from the exertion. I'm crouched down and looking at Kurt face to face:*

*Kurt: I defended number 5.*

*James: What's the problem?*

*David: He defends number 6, and he can't stop him, and he doesn't want number 13.*

*Rob: Number 6 was mine.*

*James: We can't spend the whole period talking about who is defending who.*

*Rob: Tell James that number 6 was mine.*

*David- Not 6, 13.*

*Tony- The guard is number 13, he is the one Cooper should defend.*

*Brian: Cooper's, mate.*

*There is a dance of numbers and responsibilities that I am not quite clear about. What is clear is that there is a player, who has scored the last four counterattack points against us, that has been alone, has had no defence. In the end I ask:*

*James: We can't spend the whole time arguing about who has one and who has the other. Number 13, 13 hurt us a lot, who had number 13?*

*David (and others): Kurt.*

*And as I see that Kurt assumes responsibility, I also see a spark in his eyes. His eyes fill with tears because of how badly he feels having failed in his responsibility. When I see this spark, I am disoriented: Is it Kurt? The one who is always joking? The carefree one? I never thought that spark would happen. But it did.*

*I see his grief for having failed the team (or perhaps for being reprimanded by his teammates). I prefer not to rub salt into the wound and excuse him, while thoughts of compassion whirl around: “There's no point reproaching him more”, “With the beating they are giving us it's not worth it”, “Poor guy, he's doing the best he can…”.*

*James: It was yours, well …* *He thought he had number 5, right?*

*David: We already told him.*

*James: Well, it doesn't matter, it doesn't matter. What we mustn't do is spend half the time arguing about it while they make counterattacks, right? So, what do you think?*

*Larry: Terrible.*

In this story, the coach is concerned about the team's defensive vulnerabilities and wants to address them efficiently, so the players engage in a discussion about defensive responsibilities during the half-time break. This aspect embodies the category of individual accountability of CL (8). Rather than allowing players to express themselves spontaneously, James now narrows the conversation towards a positive atmosphere, showing a change from his behaviour at the beginning of the season. Coach James directs the conversation to the need for the players to accept and respect other's faults. Again, the way coach James reflects upon the scenario and guides the conversation defines the learning zone and helps players to internalise (17) teamwork.

It is May and the last game of the season arrives, and with this last story we can illustrate period 3 (improvements in the meaning of teamwork). During the previous months, the coach had placed efforts in defining the learning zone by setting clear guidelines to discuss teamwork with the young athletes in a positive atmosphere. He did it with a CL approach, and at the end of the season the learning outcomes in players was noticeable, demonstrating how they had internalised the coach's meaning of teamwork. It can be seen by the positive presence of the five basic CL conditions in the whole conversation. This story exhibits a celebratory and light-hearted atmosphere after the last game of the season.

### Story 5. Coach to the shower!

*Today is the last game of the season, and what's at stake is not being at the bottom. In the initial talk of the match, I make them think and say what we need to do in order to win. Technical, tactical and attitudinal aspects arise. The game is played with maximum intensity and in the end we win. The guys return tired and happy to the* locker room*.*

*Steve: We broke statistics and scored more points than usual. They scored very few points, we defended brilliantly!*

*Rob: We played really well guys. Some blocks were perfect, and we managed to make quite a lot of baskets.*

*James: Good game guys, well done!*

*Oscar: I'm sorry, I failed in the defensive balance, I didn't go down fast enough and we were counterattacked.*

*James: Tony? Anything to say?*

*Tony: I missed a pass after getting a rebound which cost us 2 points from the opposing team. Sorry.*

*James: Yes, yes, these balls are very important. After a rebound we must secure the next passes. You have to improve it* *…* *And how did the opponents do?*

*Xavier: They were very aggressive, but we kept them at bay.*

*Brian: I'm not going to say anything.*

*James: You're not going to say anything? Why not?*

*Brian: Because if I say it, if I say it …* *Well … in short, when we wanted to be aggressive, they called a foul. I know you'll get angry but … It's true. I threw myself at a ball and he said I was kicking. I threw myself at another ball, I grabbed it, they hit me, and it was my foul.*

*James: Good! Very good! Now you've said it. Let's move on.*

*Larry: I didn't care if I won or lost. I mean, to be at the bottom.*

*James: I don't agree with this attitude. So Larry, why do you play basketball? You won't always win, you know.*

*In this moment I try to give a final speech/debate about the importance of teamwork, of personal improvement, of helping each other … but when the guys see where I'm going, they realise it and the conversation drifts towards a chat. They are fed up with my waffle!*

*Rob: Shall we keep James?*

*James: Do you want to say something else?*

*Kurt: Coach to the shower! Coach to the shower!*

*All together: James to the shower! James to the shower! Yeeeeaaaahhhhhh (shouting and laughing).*

*And they throw me in the shower. And one by one we throw ourselves in. All together within the four walls, soaking wet, jumping and shouting and laughing**,** washing away everything we have been through during this season that is ending. My concerns, my doubts and my worries wash away for a moment with the water. I am convinced that this is the moment I have to seek as a coach, my great success!*

*Kurt: Get wet coach, you're becoming boring with so much reflection.*

What can be observed from this story is that face-to-face promotive interaction (8) emerges when the players are encouraged and the conversation produces a climate of interpersonal skills respecting each person's turn to speak and listening to the teammates. The players engage in some post-game reflection, sharing both positive aspects and areas for improvement from the match, which results in a significant change in the meaning of teamwork. Some players admit to specific mistakes or acknowledge the aggressiveness of the opponents but emphasise that the team managed to keep them at bay. It shows how the players have reshaped the meaning of the same events they experienced during the beginning of the season, making sense of the world (21) with respect to teamwork differently. There is still frustration with the referees' decisions, but Brian's reflection is completely different from the first conversations of the season. With respect to his “functions” as an individual player in relation to the team objective (20) he now assumes full responsibility. Coach James attempts to provide a final speech on the importance of teamwork and personal improvement, but the players playfully divert the conversation into a chat. Despite the playful chaos, for James this is a moment of great success as a coach, reinforcing the importance of language as a powerful aid available to promote the learning (18) of teamwork in youth sport.

## Discussion

In summary, the five stories have permitted us to understand how conversations could be interpreted in the form of the five CL categories proposed by Johnson et al. (8). Overall, the autoethnographic storytelling highlights the complexity to learn to teamwork because of the singularities and complexities of a single youth team. Rather than merely prescribed, learning to teamwork is shown as a complex, singular and contextual that depends on the interpretation process. The results lead us to two essential contributions to be discussed from a sociocultural perspective:

### Youth team sports coaching requires the establishment of clear boundaries for the athlete to identify the learning pathway

The first contribution sheds light on how the coach acts as an expert by providing support and guidance to the players through communication. The aids (17) applied by the coach during the games' conversations shown in the selected stories align with Vygotsky's zone of proximal development (5) framework by emphasising collaborative learning, social interactions and the role of the expert in facilitating the cognitive development (19) of the players. This development is embodied in a better meaning of teamwork (2) and evidenced by players' statements in dialogues. Three actions for scaffolding (21) the improvement in the players' understanding, as a cultural learning process (18), of teamwork were carried out: a) narrowing the dialogue boundaries to a positive team atmosphere; b) focusing on CL approach categories as conversational topics of interest; and c) giving examples of appropriate and respectful ways to communicate with others. All these coaching aids framed the players' learning zone (5) and enabled them to identify with accuracy what players should think and say and what the players should do while playing. This defines the learning pathway. The ability of the coach to tune his thoughts and communication to each individual and unexpected conversations, reframed the learning zone throughout the unforeseen dialogues. In conclusion, despite the apparent difficulties reported in the beginning of the season regarding the players' meaning of teamwork, the aids used by the coach James contributed to their internalising a better understanding of teamwork within the zone of proximal development.

### The cooperative learning approach significantly contributes to the development of teamwork in youth sport

The starting point of the research is the will of a coach to improve his coaching communication strategies (4) by adopting an educative and democratic approach based on CL, as opposed to the authoritarian coaching style James experienced as a young athlete. During the storyline, as well as throughout the season, the players' understanding teamwork is steadily reshaped by the introduction of the five basic conditions of CL (8). The research also provides a real-world example of learning problems and their potential solutions for coaching with an educative and democratic approach. Each of the basic CL conditions frames an accurate and positive meaning of teamwork and showcases the teammates' internalisation (17) of it. Therefore, the results support the idea that to promote teamwork, it is not necessary to take an authoritarian coaching approach; clear boundaries can still be set by adopting an educative and democratic coaching approach. In particular, the CL approach contributes to developing personal and social skills (14) for positive participation in a team sport, even in a very competitive setting such as a youth team sport.

## Conclusions

The aim of this article was to show, from a sociocultural perspective on communication and cooperative learning, how teamwork is fostered in youth sport. The results are presented through five stories that demonstrate the complexity of communication in a competitive context and the coach's educational desire to foster teamwork among his players. As limitations, we should acknowledge that putting the focus on conversations hinders the process of collecting and coding them. In particular, it should be recognized that conversations do not represent the whole communication setting, as they cannot showcase the full context and lose the contextual nuances of non-verbal behaviour, or the tone and volume of James and team players' voices. Furthermore, transcription is sequential, but conversations have inherent overlaps or interruptions made by the athletes during the discussion. This may constrain the interpretation of certain interactional dynamics.

Nonetheless, the autoethnographic narrative research in this study brings to light significant aspects of youth sport. Specifically, thanks to the applied research design, we have been able to introduce the research in the significant real-world environment of a locker room, which is a place that is greatly restricted and protected by coaches. This is significant because most of these moments are kept completely hidden from anyone who is not part of the team, including researchers. The conversations show us the individual and collective experiences of learning in youth sport teamwork; and the research has also allowed us to unveil the thoughts of a coach dealing with the complexities of making youth athletes play as a team. Unlike research approaches that reduce the complexity to the extent that the research context does not represent the real-world, this research is based on the uncertain, complex and singular scenario that any coach faces. We modestly believe that this research realistically portrays challenges and potential solutions encountered in coaching youth sports and offers a valuable case study to use as an example of how teamwork evolves and how applying CL approach to coaching can contribute to developing teamwork in youth teams.

## Data Availability

The transcripts, field notes, and coding generated and analyzed for the study are securely stored by the corresponding author and are available upon request.

## References

[1] Lyle J. Sports Coaching Concepts: A Framework for Coaches’ Behaviour. London: Routledge (2002).

[2] McEwan D Beauchamp MR. Teamwork training in sport: a pilot intervention study. J Appl Sport Psychol. (2020) 32(2):220–36. 10.1080/10413200.2018.1518277

[3] Bloom GA Loughead TM Newin J. Team building for youth sport. J Phys Educ Recreat Dance. (2008) 79(9):44–7. 10.1080/07303084.2008.10598246

[4] Martens R. Successful Coaching. Champaign, IL: Human Kinetics (2004).

[5] Vygotsky LS. Thought and Language. Cambridge, MA: MIT Press (1986).

[6] Smith B. Narrative analysis in sport and exercise: how can it be done? In: Smith B Sparkes AC, editors. Routledge Handbook of Qualitative Research in Sport and Exercise. London: Routledge (2016). p. 282–95.

[7] Dewey J. Democracy and Education: An Introduction to the Philosophy of Education. New York: The Free Press (1966).

[8] Johnson DW Johnson RT Holubec EJ. Cooperative Learning in the Classroom. Virgina: Association For Supervision and Curriculum Development (1994).

[9] Kagan S. Cooperative Learning;. San Juan Capistrano, CA: Kagan Cooperative Learning (1994).

[10] Dyson B Casey A. Cooperative Learning in Physical Education and Physical Activity : A Practical Introduction. London: Routledge (2016).

[11] Dyson BP Linehan NR Hastie PA. The ecology of cooperative learning in elementary physical education classes. J Teach Phys Educ. (2010) 29(2):113–30. 10.1123/jtpe.29.2.113

[12] Metzler M Colquitt G. Instructional Models for Physical Education. 4th ed. New York: Routledge (2021). 10.4324/9781003081098

[13] Slavin RE. Cooperative learning and academic achievement: why does groupwork work?. [Aprendizaje cooperativo y rendimiento académico: ¿por qué funciona el trabajo en grupo?]. An Psicol Ann Psychol. (2014) 30(3):785–91. 10.6018/analesps.30.3.201201

[14] Casey A Goodyear VA. Can cooperative learning achieve the four learning outcomes of physical education? A review of literature. Quest. (2015) 67:56–72. 10.1080/00336297.2014.984733

[15] Leo FM González-Ponce I López-Gajardo MA Pulido JJ García-Calvo T. Team building intervention program and its relationship with group processes in young athletes. Int J Sport Psychol. (2021) 52:120–36. 10.7352/IJSP.2021.52.120

[16] Moen T. Reflections on the narrative research approach. Int J Qual Methods. (2006) 5(4):56–69. 10.1177/160940690600500405

[17] Vygotsky LS. Mind in Society: The Development of Higher Psychological Processes. Cambridge MA: MIT Press (1978).

[18] Cole M. Cultural Psychology: A Once and Future Discipline. Cambridge, MA, US: Harvard University Press (1996).

[19] Edwards D Mercer N. Common Knowledge: The Development of Understanding in the Classroom. London: Routledge (2012). 10.4324/9780203095287

[20] Soltanzadeh S Mooney M. Players within a team: understanding the structure of team performance through individual functions and team objectives. Int Sport Coach J. (2018) 5(1):84–9. 10.1123/iscj.2017-0032

[21] Bruner JS. Acts of Meaning. London: Harvard University Press (1990).

[22] Braun V Clarke V. Reflecting on reflexive thematic analysis. Qual Res Sport Exerc Health. (2019) 11(4):589–97. 10.1080/2159676X.2019.1628806

[23] Dowling F Garrett R. Narrative inquiry and research on physical activity, sport and health: exploring current tensions. Sport Educ Soc. (2016) 21(1):1–6. 10.1080/13573322.2015.1112262

[24] Richardson L. Getting personal: writing-stories. Int J Qual Stud Educ. (2001) 14(1):33–8. 10.1080/09518390010007647

[25] Richardson L St. Pierre EA. Writing: a method of inquiry. In: Denzin NK Lincoln YS, editors. The Sage Handbook of Qualitative Research. Thousand Oaks, CA: Sage Publications Ltd. (2005). p. 959–78.

[26] Sparkes AC. Autoethnography: accept, revise, reject? An evaluative self reflects. Qual Res Sport Exerc Health. (2020) 12(2):289–302. 10.1080/2159676X.2020.1732453

[27] Smith B Sparkes AC. Narrative analysis and sport and exercise psychology: understanding lives in diverse ways. Psychol Sport Exerc. (2009) 10(2):279–88. 10.1016/j.psychsport.2008.07.012

[28] Bowles R O’Dwyer A. Athlete-centred coaching: perspectives from the sideline. Sports Coach Rev. (2020) 9 (3):231–52. 10.1080/21640629.2019.1649901

[29] Carless D Douglas K. Stories as personal coaching philosophy. Int J Sports Sci Coach. (2011) 6 (1):1–12. 10.1260/1747-9541.6.1.1

[30] Rathwell S Callary B Young B. Exploring the context of coached masters swim programs: a narrative approach. Int J Aquat Res Educ. (2015) 9(1):7. 10.25035/ijare.09.01.07

[31] Arumí-Prat J Torres-Cladera G Pla-Campas G. “En El Buen Camino”: Colaboración Entre Maestros e Investigadores Para La Implementación Del Aprendizaje Cooperativo En La Educación Física de Educación Primaria. J Teach Phys Educ. (2024) 44:174–81. 10.1123/jtpe.2024-0086

[32] Middleton TRF Wadey R Cavallerio F Wagstaff CRD Sparkes AC. Telling tales in sport, exercise, and performance psychology: the how, what, and why of creative analytical practices. Sport Exerc Perform Psychol. (2025) 14(1):160–74. 10.1037/spy0000363

[33] Clandinin DJ Pushor D Orr AM. Navigating sites for narrative inquiry. J Teach Educ. (2007) 58(1):21–35. 10.1177/0022487106296218

